# Healthcare Workers’ Knowledge and Resource Availability for Care of Sickle Cell Disease in Dar es Salaam, Tanzania

**DOI:** 10.3389/fgene.2021.773207

**Published:** 2022-02-11

**Authors:** Agnes Jonathan, Hilda Tutuba, William Lloyd, Joyce Ndunguru, Julie Makani, Paschal Ruggajo, Irene K. Minja, Emmanuel Balandya

**Affiliations:** ^1^ Sickle Pan-African Research Consortium (SPARCO)-Tanzania, Dar es Salaam, Tanzania; ^2^ Sickle Cell Program, Department of Hematology and Blood Transfusion, MUHAS, Dar es Salaam, Tanzania; ^3^ Department of Internal Medicine, MUHAS, Dar es Salaam, Tanzania; ^4^ Department of Restorative Dentistry, MUHAS, Dar es Salaam, Tanzania; ^5^ Department of Physiology, MUHAS, Dar es Salaam, Tanzania

**Keywords:** sickle cell disease, knowledge, healthcare workers, resources, health facilities, SPARCO, Tanzania

## Abstract

**Background:** Sickle cell disease (SCD) is a global public health priority due to its high morbidity and mortality. In Tanzania, SCD accounts for 7% of under-five mortality. Cost-effective interventions such as early diagnosis and linkage to care have been shown to prevent 70% of deaths but require knowledge among healthcare workers and availability of resources at health facilities. In Tanzania, data on these critical determinants are currently lacking.

**Objective:** To assess healthcare workers’ knowledge and resource availability for care of SCD at health facilities in Dar es Salaam, Tanzania.

**Methodology:** A facility-based cross-sectional study was conducted between December 2020 and February 2021 among 490 nurses and clinicians at Regional Referral Hospitals (Temeke, Amana, and Mwananyamala) and Muhimbili National Hospital in Dar es Salaam, Tanzania. Data were collected using a pre-tested structured questionnaire consisting of 13 knowledge questions (scored good knowledge if correct response in >7) and an inventory check list to record available resources. Pearson’s χ^2^ was used to determine the association between level of knowledge and demographic factors. Multivariate logistic regression was used to ascertain the strength of associations. A two-tailed *p*-value <0.05 was considered to be statistically significant.

**Results:** Of the 490 participants (median age 28 years [IQR = 26–35]), only 25.1% had good knowledge on SCD. The odds of good knowledge was 82% lower in nurses than clinicians (AOR = 0.177; 95% CI: 0.090, 0.349; *p* < 0.001); 95% lower in diploma than Master’s degree holders (AOR = 0.049; 95% CI: 0.008, 0.300; *p* = 0.001) and 4.6 times higher in those with 5–9 years than ≥10 years of experience (AOR = 4.564; 95% CI: 1.341, 15.525; *p* = 0.015). The regional-level hospitals lacked diagnostic tests and hydroxyurea therapy.

**Conclusion:** There was general lack of knowledge on SCD among healthcare workers and limited availability of critical resources for the diagnosis and care of SCD, especially at regional-level hospitals. Efforts are needed for their improvement to enhance care to patients, thus reducing the morbidity and mortality due to SCD in Tanzania.

## Introduction

Sickle cell disease (SCD) is an inherited disorder of the hemoglobin (Hb) molecule of the red blood cells (RBCs) that is associated with serious complications and reduced life expectancy (Tluway and Makani 2017). Globally, there are >300,000 births/year of children with SCD (Piel et al., 2013). Over 75% of people with SCD live in Sub-Saharan Africa (SSA), and this proportion is projected to increase to 85% by the year 2050 (Makani et al., 2011). WHO declared SCD a public health priority in 2006 (WHO, 2006) and is part of the Tanzania National Non-communicable Disease Strategy (Ministry of Health and Social Welfare, 2015). In Tanzania, about 11,000 babies are born with SCD/year, ranking 5th in the world behind Nigeria, Democratic Republic of Congo (DRC), Angola, and India (Nkya et al., 2019). Unlike high-income countries, SCD has a higher mortality in SSA. In Tanzania, mortality rate due to SCD is 1.9 per 100 person-years of observation, contributing 7% of under-five mortality (Makani et al., 2018; Makani et al., 2011).

Studies in high-income countries have indicated that simple, cost-effective interventions such as early identification of SCD patients by newborn screening and the subsequent provision of comprehensive care can prevent 70% of deaths due to SCD (WHO, 2006). Comprehensive care includes prompt treatment of acute events, prophylaxis against infections with oral penicillin, and vaccination against *Streptococcus pneumoniae*, plus prompt diagnosis and treatment of complications and educative programs provided to all individuals with SCD (Makani et al., 2018).

Following definitive diagnosis, proper management of SCD requires healthcare workers to have appropriate knowledge of the disease, especially risk factors for complications, symptoms and signs, investigations, and appropriate interventions (Dennis-Antwi et al., 2008; Gomes et al., 2015; Janerrete et al., 2016). However, limited knowledge among implementers is one of the major reasons for poor performance of healthcare interventions (Ambrose et al., 2020), likely contributing to high childhood mortality of 50–90% among patients with SCD in SSA (Makani et al., 2018).

Few studies have investigated on the level of knowledge on SCD among healthcare workers. In the USA, 67% of physicians knew that SCD can be detected early through newborn screening (McWalter et al., 2011)**.** Another study in the USA showed 39% of obstetric-gynecologists were confident in managing pregnant women with SCD after receiving adequate training (Azonobi et al., 2014). In Brazil, approximately 75% of healthcare workers were found to have suboptimal knowledge on management of SCD (Gomes et al., 2011). In Nigeria, the knowledge of healthcare workers at primary health facilities was very poor, whereas only 37.9% had good knowledge on SCD diagnosis and crisis prevention (Adegoke et al., 2018). In the DRC, 80% of physicians knew how to diagnose SCD while 44% followed recommendation on management of vaso-occlusive crisis (VOC) while prescribing analgesia and hydration (Mbiya et al., 2020). In Tanzania, the level of knowledge on diagnosis and treatment of SCD among healthcare workers is not known.

For proper care of SCD, resources should be available at health facilities for the diagnosis and care of SCD patients (Dennis-Antwi et al., 2008). In high-income countries, the resources for diagnosis, treatment, and care of SCD are available (Drive and Quinn, 2017). In low- and middle-income countries, few studies have explored availability of resources for care of SCD showing widespread limitation where all too often resources are limited to private facilities and beyond the reach of the majority who would benefit (Williams, 2015; Mcgann et al., 2018). In the DRC, 65% of the physicians experienced difficulty in performing Hb electrophoresis due to lack of equipment (Mbiya et al., 2020). In Tanzania, the data on the availability of resources for the diagnosis and treatment of SCD appropriate to the level of healthcare facilities are not known.

This study sought to determine the level of knowledge on SCD among healthcare workers and availability of resources at Regional Referral Hospitals and Muhimbili National Hospital in Dar es Salaam for the diagnosis and care of SCD.

## Materials and Methods

### Study Design and Setting

This was a facility-based cross-sectional study conducted from December 2020 to January 2021 at Regional Referral Hospitals (Mwananyamala, Amana, and Temeke) and Muhimbili National Hospital (Upanga and Mloganzila) in Dar es Salaam, Tanzania. These hospitals are public and provide outpatient and inpatient SCD services to majority of SCD patients in the city.

All the hospitals included in this study run dedicated pediatric and adult SCD outpatient clinics, which are operated by medical doctors and nurses. Specialist pediatricians, internal medicine physicians, and hematologists are also part of care when available. In case of admissions, patients are taken care of in general pediatric, internal medicine, general surgery, and or other wards as appropriate.

Non-governmental organizations play a pivotal role in increasing public awareness and advocacy for SCD in Tanzania, and are an important bridge between health systems, researchers, and patient communities. Particularly, the study team at Sickle Cell Programme, Muhimbili University of Health and Allied Science (MUHAS) has worked closely with Tanzania Sickle Cell Disease Alliance (TANSCDA), Sickle Cell Disease Patients Community of Tanzania (SCDPCT), Sickle Cell Youth Foundation (SYF), and Tanzania Sickle Cell Warriors (TASIWA). These have facilitated fundraising for resources at health facilities, the conduct of public awareness campaigns as well as provision of health education to patients and caregivers of individuals with SCD.

### Study Participants

Healthcare workers comprising clinicians and nurses providing services and directly interacting with clients who are seeking medical services for SCD at selected facilities were eligible to participate in this study.

### Sample Size

The minimum required sample size of 416 healthcare workers was calculated using the Cochran formula (Cochran, 1977), assuming 37.9% of healthcare workers have good knowledge on SCD (Adegoke et al., 2018) and accounting for 15% non-response rate. Participants were then selected via multistage sampling. In the first stage, the participating hospitals, the Regional Referral Hospitals (Mwananyamala, Amana, and Temeke) and Muhimbili National Hospital (Upanga, Mloganzila), were identified. In the second stage, we identified the participating departments (obstetrics and gynecology, pediatric, internal medicine, surgery, and outpatient department) within the hospitals. In the third stage, the number of participants (clinicians and nurses) from each department in each hospital was determined proportionally by considering their total number at particular hospitals. Finally, the individual participants at each department were recruited consecutively whereby each available healthcare worker who met the inclusion criteria was enrolled until the sample size for each cadre was met. Also, 74 more healthcare workers were added beyond the minimum sample size for a final total of 490 participants.

### Study Variables

The dependent/outcome variables were the overall level of knowledge on SCD among healthcare workers and availability of resources for diagnosis and treatment of SCD at healthcare facilities. Independent variables were age (years), sex (male/female), duration since graduation (years), level of facility (regional referral hospital/national hospital), professional cadre (nurse officer/registered nurse/nurse midwife for nurses, assistant medical officer/medical doctor/specialist for clinicians), duration of practice (years), and short course training received on SCD (yes/no).

### Data Collection

Data on the sociodemographic characteristics (age, sex, level of education, duration since graduation, years of practice, level of facility, professional cadre, and short course training on SCD) and level of knowledge on SCD (overall, diagnosis and treatment) were obtained using a pre-tested and validated self-administered questionnaire adapted from Brazil (Diniz et al., 2019a) and modified to fit local context. Questions on SCD covered knowledge on diagnosis disease genotype, ideal timing of screening for SCD, confirmatory tests for SCD, clinical features of SCD, conditions that favor sickling of RBCs in patients with SCD warning signs in SCD, and knowledge on treatment of SCD (management of acute complications of SCD, drugs used to treat pain crises in patients with SCD, drugs used to prevent and/or treat complications of SCD, indications for use of antibiotics in SCD, indications for blood transfusion to patients with SCD, means of preventing infections in patients with SCD as well as pregnancy, and use of contraception in patients with SCD). The questions were in a multiple-choice setup and participants had to choose one best response for each question. Data on resources available at the facilities were obtained by inventory checklist adapted from the Tanzania National Guideline for SCD Management (United Republic of Tanzania, 2020) and Basic Health Standard for Facilities in Tanzania (United Republic of Tanzania, 2017) that indicate the facility level-specific minimum required resources through observation method. In each facility, one checklist was filled by interrogating heads of relevant units and cross-checking the mentioned drug (antibiotics and antimalarials, anti-pain medication, folic acid, and hydroxyurea) whether available and not yet expired, equipment (diagnostic equipment, other laboratory investigations, imaging equipment, and point-of-care clinical tests) whether available and functional, blood transfusion and exchange transfusion services, emergency surgical services, and intensive care unit whether available or not. Expired drugs and non-functioning instruments were regarded as not available.

### Statistical Analysis

Data were checked for their completeness and consistence before analysis. Open-ended questions in the demographics section were first edited, categorized, and coded (level of education was categorized into certificate, diploma, degree, and Master’s level) before entry. Participants’ characteristics were first analyzed using descriptive statistics. Continuous variables (age, duration since graduation, and years of practice) were tested for normality of distribution using Shapiro and Wilk test. All variables were found to be not normally distributed, so they were summarized in median and interquartile ranges, then grouped into different categories as follows: age (0–30, 31–40,41–50, and 51–60 years), duration since graduation (≤5, >5 years), and years of practice (0–4, 5–9, >10 years). These, together with other categorical variables (sex, level of education, level of facility, professional cadres, and short course training received on SCD), were summarized in frequencies and percentages and presented in tables.

Knowledge on SCD was assessed by asking 13 multiple-choice questions comprising 6 questions on diagnosis and 7 questions on treatment. For each question, 1 point was assigned to each correctly answered item and 0 points otherwise. For calculation of the overall knowledge on SCD, the sum of all correct answers for diagnosis and treatment was taken, considering the following knowledge score ranges: >7 (more than 54% accuracy), good; ≤7 (54% or less accuracy), poor (Diniz et al., 2019a).

Inferential statistics on factors associated with level of knowledge was done by comparing the different categories between the dependent variable (overall knowledge on SCD) with various independent variables (age, sex, level of education, duration since graduation, level of facility, professional cadres, years of practice, and short course training received on SCD) using χ^2^ test (for independent variables with all expected values ≥5). Two-tailed *p*-values below 0.05 were considered statistically significant. Subsequently, univariate and multivariate binomial logistic regression were done to test the strength of association between the main dependent variable (overall level of knowledge) with the independent variables. Only independent variables with *p*-values ≤0.2 in univariate analysis (age, sex, professional cadres, duration since graduation, level of education, and years of practicing) were entered into multivariate logistic regression to adjust for effect of multiple predictors. The odds ratio was used to explain the relationship between dependent variable and associated factors, and confidence level at 95% as well as *p*-values were used in determining statistical significance.

In analyzing availability of resources for diagnosis and care of SCD, descriptive statistics were used to summarize the resources available at all health facilities in frequencies and percentages and presented in table and bar chart. Data were analyzed using IBM SPSS Statistics for Windows version 23.0 (IBM Corp., Armonk, NY, USA).

### Ethical Considerations

Approval for the study was obtained from the Muhimbili University of Health and Allied Sciences (MUHAS) Research Ethics Committee with ethical clearance number MUHAS-REC-12-2020-452. Permission to conduct the study was sought from executive directors of relevant municipal hospitals and Muhimbili National Hospital. Written informed consent was obtained from all participants before the questionnaires were answered.

## Results

### Demographic Characteristics of Participants

A total of 490 healthcare workers comprising 46.7% (229/490) nurses and 53.3% (261/490) clinicians completed the questionnaires. As shown in Table 1, the age of participants ranged from 23 to 60 years with the median age of 28 years [IQR = 26–35]. The majority of the participants were female 54.3% (266/490); 70.2% (334/490) had graduated ≤5 years; 64.3% (315/490) had less than 5 years of practicing experience and 76.3% (374/490) had one or two university degrees. Out of 490 participants, 173 (35.3%) worked at Regional Referral Hospitals and 317 (64.7%) at National Hospital. Only 8.2% had received short course training on SCD.

**TABLE 1:** Socio-demographic characteristics and overall level of knowledge on SCD among healthcare workers in Dar es Salaam (N = 490)

Characteristic	*n* (%)	Overall knowledge on SCD		
		Poor (≤7) N = 367 *n* (%)	Good (>7) N = 123 *n* (%)	*p*-value (χ^2^ test)
Age (years), median = 28 [IQR = 26–35]				0.083
21–30	315 (64.3)	234 (74.3)	81 (25.7)	
31–40	116 (23.7)	82 (70.7)	34 (29.3)	
41–50	38 (7.8)	31 (81.6)	7 (18.4)	
51–60	21 (4.3)	20 (95.2)	1 (4.8)	
Sex				0.104
Male	224 (45.7)	160 (71.4)	64 (28.6)	
Female	266 (54.3)	207 (77.8)	59 (22.2)	
Duration since graduation (years)				0.002
≤5	344 (70.2)	244 (70.9)	100 (29.1)	
>5	146 (29.8)	123 (84.2)	23 (15.8)	
Level of education				0.000
Certificate	29 (5.9)	29 (100)	0	
Diploma	87 (17.8)	85 (97.7)	2 (2.3)	
Degree	335 (68.4)	241 (71.9)	94 (28.1)	
Masters	39 (8)	12 (30.8)	27 (69.2)	
Name of facility				
Mwananyamala RRH	50 (10.2)	39 (78)	11 (22.0)	0.813
Temeke RRH	56 (11.4)	43 (76.8)	13 (23.2)	
Amana RRH	67 (13.7)	52 (77.6)	15 (22.4)	
Muhimbili National Hospital	317 (64.7)	233 (73.5)	84 (26.5)	
Level of facility				0.335
Regional Referral Hospital	173 (35.3)	134 (77.5)	39 (22.5)	
Muhimbili National Hospital	317 (64.7)	233 (73.5)	84 (26.5)	
Professional cadre				0.000
Nurses	229 (46.7)	215 (93.9)	14 (6.1)	
Clinicians	261 (53.3)	152 (58.2)	109 (41.8)	
Years of practice (years)				0.010
<5	315 (64.3)	235 (74.6)	80 (25.6)	
5–9	75 (15.3)	48 (64)	27 (36)	
≥10	100 (20.4)	84 (74.9)	16 (25.1)	
SCD training received				0.988
No	450 (91.8)	30 (75.0)	10 (25)	
Yes	40 (8.2)	337 (74.9)	113 (25.1)	

### Overall Level of Knowledge on SCD Among the Study Participants

Only 25.1% (123/490) of healthcare workers had good knowledge on SCD. In ascertaining association between the overall level of knowledge on SCD with participants’ characteristics, it was observed that the duration since graduation, level of education, professional cadre, and years of practice were significantly associated with the level of knowledge on SCD (Table 1).

### Regression Analysis of Factors Influencing Knowledge on SCD Among Healthcare Workers

Table 2 summarizes results of univariate and multivariate logistic regression. The final model revealed that there was strong association between overall level of knowledge on SCD with level of education, professional cadres, and years of practice. The odds of nurses having good knowledge on SCD were 82% lower than that in clinicians (AOR = 0.177; 95% CI: 0.090, 0.349; *p* < 0.001). Furthermore, healthcare workers with diploma had 95% lower odds of having good knowledge on SCD compared with those with Master’s degree (AOR = 0.049; 95% CI: 0.008, 0.300; *p* = 0.001). Likewise, those with university degree had 72% lower odds of having good knowledge of SCD compared with those with Master’s degree (AOR = 0.284; 95% CI: 0.0960, 0.837; *p* = 0.022). The healthcare workers with 5–9 years of practice were 4.6 times more likely to have good knowledge on sickle cell disease than those with practicing experience of 10 years and above (AOR = 4.564; 95% CI: 1.341, 15.525; *p* = 0.015**)**.

**TABLE 2:** Regression analysis of factors influencing knowledge among healthcare workers

Factors	*n* (%)	Univariate analysis		Multivariate analysis	
		COR (95% CI)	*p*-value	AOR (95% CI)	*p*-value
Age (years)					
21–30	315 (64.3)	6.923 (0.915–52.408)	0.061	0.343 (0.029–4.112)	0.398
31–40	116 (23.7)	8.293 (1.070–64.273)	0.043	0.374 (0.032–4.339)	0.431
41–50	38 (7.8)	4.516 (0.516–39.529)	0.173	0.484 (0.041–5.701)	0.564
51–60	21 (4.3)	Reference			
Sex					
Male	224 (45.7)	1.403 (0.932–2.114)	0.105	0.969 (0.603–1.556)	0.896
Female	266 (54.3)	Reference			
Duration since graduation (years)					
≤5	344 (70.2)	2.192 (1.326–3.622)	0.002	1.231 (0.514–2.948)	0.641
>5	146 (29.8)	Reference			
Level of education					
Certificate	29 (5.9)	0.00 (0.00–)	0.998	0.000 (0.000–)	0.998
Diploma	87 (17.8)	0.01 (0.002–0.050)	0.002	0.049 (0.008–0.300)	0.001
Degree	335 (68.4)	0.173 (0.084–0.356)	0.005	0.284 (0.096–0.837)	0.022
Masters	39 (8)	Reference			
Level of Facility					
Regional RRH	173 (35.3)	0.807 (0.522–1.247)	0.335		
National hospital	317 (64.7)	Reference			
Professional cadre					
Nurses	229 (46.7)	0.091 (0.050–0.164)	0.000	0.177 (0.090–0.349)	0.000
Clinicians	261 (53.3)	Reference			
Years of practice (years)					
<5	315 (64.3)	1.787 (0.989–3.230)	0.05	1.533 (0.391–6.009)	0.540
5–9	75 (15.3)	2.953 (1.448–6.024)	0.003	4.564 (1.341–15.525)	0.015
≥10	100 (20.4)	Reference			
SCD training received					
Yes	450 (91.8)	1.006 (0.477–2.123)	0.988		
No	40 (8.2)	Reference			

COR, crude odds ratio; AOR, adjusted odds ratio; 95% CI, confidence interval at 95%.

### Resources Available for Diagnosis and Treatment of SCD

Table 3 shows the resources available at Regional Referral Hospitals (RRH) and Muhimbili National Hospital (MNH) for diagnosis and treatment of SCD. Out of the equipment for SCD diagnosis, only the sickling test was uniformly available at both RRH and MNH while Hb electrophoresis was only available at the MNH. Diagnostic tests such as SickleSCAN, isolectric focusing (IEF), and high-performance liquid chromatography (HPLC) were not available at both the RRH and MNH. All other relevant laboratory equipment were present at the MNH but some, such as equipment for blood and urine culture, and were only present in 33.3% of the RRH. The point-of-care clinical resources used at the clinics such as BP machine, pulse oximeter, stethoscope, and thermometer were widely available at both the RRH and MNH. All the imaging equipment including X-ray, ECG, echocardiography (ECHO), trans-cranial Doppler ultrasound (TCD), CT scan, and MRI were available at the national level. On the other hand, at regional level, on X-ray machines were uniformly present while ECG, ECHO, and TCD were only present in some RRH while CT scan and MRI machines were not available.

**TABLE 3:** Resources available at healthcare facilities in Dar es Salaam

S/N	Category of resource	Item	Availability	
			RRHs	MNH
1	Equipment for SCD diagnosis	Sickling test	3/3 (100)	1/1 (100)
		Isoelectric focusing (IEF)	0/3 (0)	0/1 (0)
		Hb electrophoresis	0/3 (0)	1/1 (100)
		HPLC	0/3 (0)	0/1 (0)
		Point-of-care tests (e.g., SickleSCAN)	0/3 (0)	0/1 (0)
2	Other laboratory investigations	Hematology analyzer	3/3 (100)	1/1 (100)
		Peripheral blood smear	1/3 (33.3)	1/1 (100)
		Biochemistry analyzer	3/3 (100)	1/1 (100)
		Blood culture	1/3 (33.3)	1/1 (100)
		Urine culture	1/3 (33.3)	1/1 (100)
		Malaria rapid diagnostic test (MRDT)	3/3 (100)	1/1 (100)
		Blood grouping and cross-matching	3/3 (100)	1/1 (100)
		Erythrocyte sedimentation rate (ESR)	2/3 (66.7)	1/1 (100)
		HIV rapid test	3/3 (100)	1/1 (100)
		PCR machine	0/3 (0)	1/1 (100)
3	Point-of-care clinical tests	BP machine	3/3 (100)	1 (100)
		Stethoscope	3/3 (100)	1 (100)
		Weighing scale	2/3 (66.7)	1 (100)
		Thermometer	3/3 (100)	1 (100)
		Tape measure	1/3 (33.3)	1 (100)
		Pulse oximeter	3/3 (100)	1 (100)
		Oxygen machine	3/3 (100)	1 (100)
		Hemocue machine	3/3 (100)	1 (100)
		Dipstick urinalysis	3/3 (100)	1/1 (100)
4	Imaging	ECHO	2/3 (66.7)	1/1 (100)
		ECG	2/3 (66.7)	1/1 (100)
		Ultrasound	3/3 (100)	1/1 (100)
		TCD	1/3 (33.3)	1/1 (100)
		X-Ray	3/3 (100)	1/1 (100)
		CT machine	0/3 (0)	1/1 (100)
		MRI	0/3 (0)	1/1 (100)
5	Anti-pain medication	Paracetamol	3/3 (100)	1/1 (100)
		Ibuprofen	3/3 (100)	1/1 (100)
		Diclofenac	3/3 (100)	1/1 (100)
		Pethidine	2/3 (66.7)	1/1 (100)
		Morphine	2/3 (66.7)	1/1 (100)
6	Antibiotics and antimalarials	Penicillin V	3/3 (100)	1/1 (100)
		Amoxiclav	3/3 (100)	1/1 (100)
		Ceftriaxone	3/3 (100)	1/1 (100)
		Metronidazole	3/3 (100)	1/1 (100)
		Gentamicin	2/3 (66.7)	1/1 (100)
		Artemether lumefantrine (ALU)	3/3 (100)	1/1 (100)
7	Hydroxyurea		0/3 (0)	1/1 (100)
8	Folic acid		3/3 (100)	1/1 (100)
9	IV fluids	Normal saline/Ringer’s lactate	3/3 (100)	1/1 (100)
10	Blood transfusion and exchange transfusion	Blood transfusion	3/3 (100)	1/1 (100)
		Exchange transfusion	0/3 (0)	0/1 (0)
11	Emergency surgical and ICU services	Emergency surgical capability	2/3 (67)	1/1 (100)
		Intensive care unit (ICU)	1/3 (33)	1/1 (100)

**FIGURE 1:**
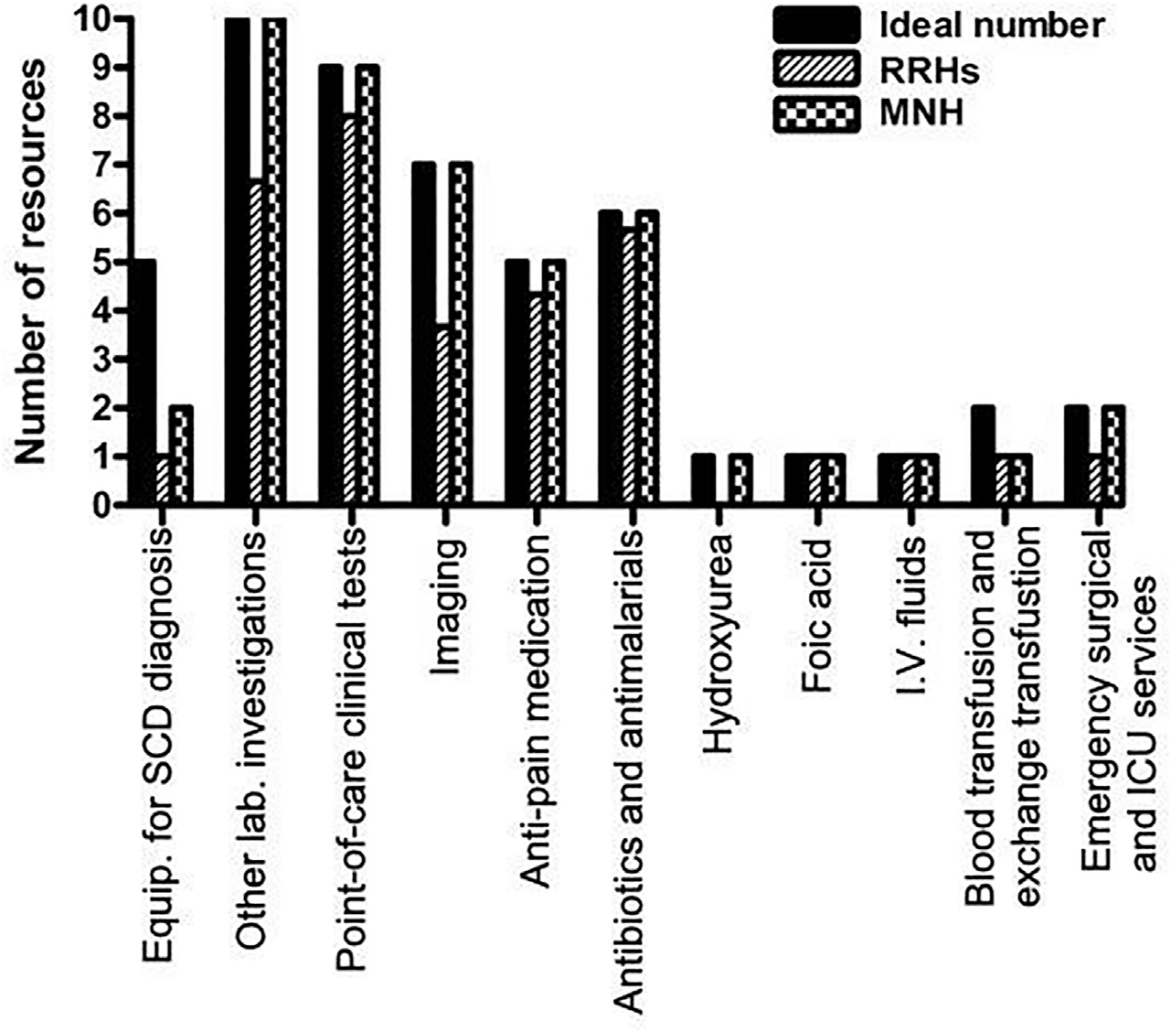
Ideal versus available resources for SCD care at healthcare facilities in Dar es Salaam.

Drugs such as folic acid, antibiotics, and antimalarials as well as painkillers (paracetamol, ibuprofen, diclofenac, and morphine) were to a large extent available at both RRH and MNH while hydroxyurea was only available at the national level. Blood transfusion services with whole blood and packed RBCs were available at all RRH and MNH, while exchange transfusion services were not available at all facilities. Capacities for emergency surgical procedures such as splenectomy and intensive care unit were available in 66.7 and 33.3% of the regional referral hospitals, respectively, while both services were available at the MNH. Figure 1 compares the ideal number of clinical resources required at the facility level with the actual number of resources available at the RRH (average) and MNH.

## Discussion

Proper management of SCD requires healthcare workers to have appropriate knowledge of the disease and healthcare facilities to be well equipped for diagnosis and care of individuals with SCD. In the present study, we uncovered substantial lack of knowledge on SCD among healthcare workers in health facilities in Dar es Salaam where only one-quarter of healthcare workers had good knowledge. This proportion is low compared with that reported in the USA where over two-thirds of healthcare workers had good knowledge on SCD (Diniz et al., 2019b). Furthermore, the knowledge was also lower compared with that of healthcare workers in West Africa, particularly in the DRC and Nigeria, where 80 and 37.9% of healthcare workers, respectively, were found to have good knowledge on the nature of the disease, recognizable features, early SCD diagnosis, ideal timing for screening, and efforts to prevent SCD crisis (Adegoke et al., 2018; Mbiya et al., 2020). The low level of education on SCD among healthcare workers may be attributed to limited coverage of SCD in training and continuing education programs. This low level of knowledge on SCD is concerning as it implies lower likelihood of healthcare workers to direct clients toward early diagnosis during infancy and link them to comprehensive care, which are paramount to reversal of morbidity and mortality due to SCD as evidenced in high-income countries (Makani et al., 2018).

Our study showed strong association between level of education, professional cadres, and years of practice with the level of knowledge on SCD among healthcare workers. Specifically, good knowledge on SCD was more common among clinicians, holders of university degrees, and those with experience in clinical practice of between 5 and 9 years. This may be explained by the differential coverage of SCD in training curricula for health sciences, which is more extensive for medical practitioners than nurses, especially at degree level and above (Adegoke et al., 2018; Jain and Lothe, 2015). While it is plausible that the increase in years of practice from below 5 years to between 5 and 9 years was associated with an increase in knowledge on SCD (Jain and Lothe 2015), the fall in level of knowledge with increasing practice experience of 10 years and above is intriguing and is different from observations made in the DRC where physicians with more than 10 years of experience had better knowledge on management of SCD compared with those with less than 10 years of experience (Mbiya et al., 2020. A potential explanation could be the lack of adequate content of SCD in the training curricula used a decade ago and beyond (Azonobi et al., 2014). In our study, only 8.2% of the participants had undertaken short courses on SCD, hence lacking statistical power to ascertain its contribution to the level of knowledge. However, previous studies in the USA, Brazil, Nigeria, and Ghana have reported significant influence of short course on knowledge on SCD (McWalter et al., 2011; Azonobi et al., 2014; Adegoke et al., 2018; Jain and Lothe, 2015).

To have good clinical outcomes in SCD, resources must be available at facilities for managing both inpatients and outpatients (Ambrose et al., 2020). While our study showed that resources were generally available at national-level hospitals, we uncovered suboptimal levels of resources for care of SCD at regional-level hospitals where items such as SCD confirmatory tests including Hb electrophoresis, isoelectric focusing, and HPLC; imaging equipment including trans-cranial Doppler ultrasound, CT scan, and MRI; blood and urine culture; intensive care services as well as hydroxyurea were uniformly or commonly missing. This may partly contribute to delayed diagnosis and subsequently poor outcome of the disease in Tanzania. The lack of confirmatory tests at healthcare facilities in Tanzania is similar to that reported in other parts of Africa such as in the DRC where two-thirds of the physicians (65%) reported difficulty in performing hemoglobin electrophoresis due to lack of equipment (Mbiya et al., 2020). In Tanzania, only sickling test was commonly available at all facilities (RRH and MNH), which is similar to other hospitals in Africa where screening for SCD is done using the sickling test and solubility test that are unreliable and cannot distinguish homozygous (Hb SS) from heterozygous (Hb AS) state (United Republic of Tanzania, 2017). Availability of SCD confirmatory tests will be a step forward toward universal newborn screening for SCD in Tanzania. Besides SCD confirmatory test, the observed lack of CT scan and MRI at regional-level hospitals is similar to that reported in other studies that showed supporting imaging equipment for diagnosis of SCD complications such as stroke are usually lacking at lower-level facilities in resource-limited countries (Ansong et al., 2013). Similarly, unlike national hospitals, other supporting tests such as urine and blood culture were not readily available at RRHs. However, point-of-care tests such as BP machines, stethoscopes, thermometers, pulse oximeters, hemocue machine, urinary dipstick, and weighing scales were commonly available at both RRH and MNH, implying capability for thorough physical examination, which is paramount during routine clinic visits (Diniz et al., 2019b).

SCD is contributing to the anemia in under-fives and pregnant women in areas of high prevalence. Severe anemia in SCD is life threatening and requires prompt treatment with blood transfusion using whole blood or packed RBCs (Tanyi, 2003). Our study showed common availability of blood transfusion services with whole blood as well as packed RBCs at all RRH and MNH. However, there were no capabilities for exchange transfusion services at all facilities at the time of the study, although such capabilities have since been established at the MNH. It is desirable to develop capabilities for exchange transfusion, which is required for treatment of life-threatening emergencies such as acute chest syndrome, at specialized referral care centers (Chamba et al., 2018).

Comprehensive care of SCD includes treatment of vaso-occlusive crisis, and prevention and prompt treatment of bacterial infections, malaria, and severe anemia. This study showed that drugs such as folic acid, antibiotics, and antimalarials and painkillers (paracetamol, ibuprofen, diclofenac, and morphine) were to a large extent available at both RRH and MNH while hydroxyurea was only available at the national level. While the trend in availability of most essential drugs is encouraging (Ambrose et al.,. 2020; Mbiya et al., 2020), it is concerning that hydroxyurea was not available at RRH. Currently in Tanzania, the RRH are allowed to procure hydroxyurea, and the medical, pediatric, and hematology specialists are allowed to prescribe hydroxyurea to patients with SCD. A major factor therefore hindering availability of hydroxyurea at RRH may be low awareness among the specialists, which leads to lower trends in prescribing the drug and consequently low rate or lack of procurement of the drug by hospital pharmacies. Efforts are required to ensure increased availability of hydroxyurea not only at MNH but also RRH.

Our study was not without limitations. Assessment of the level of knowledge on diagnosis and treatment of SCD was based on a set of 6 and 7 questions, respectively, which may not be comprehensive enough to exhaust all facets of knowledge. However, the questions used interrogated basic concepts and the number of questions used has been validated to be sufficient in evaluating the level of knowledge on SCD among healthcare workers in a previous study (Williams 2015).

In conclusion, there is a serious lack of knowledge on SCD among healthcare workers at healthcare facilities in Dar es Salaam. This calls for interventions through enhancement of the coverage of SCD as a model genetic disease in college and university training programs as well as in mandatory continuing professional development and continuing medical education programs to all staff. While intervention programs are advocated across the entire spectrum, major emphasis should be among the non-clinician cadres and non-degree holders. Further, the RRH should be equipped with vital resources to support SCD diagnosis and care, and specialized services such as exchange blood transfusion should be built at designated centers of excellence, such as the MNH, where in-need patients can be referred to, all aiming to improve the survival and quality of life of individuals with SCD in Tanzania. Similar studies should be conducted in other parts of the country, particularly in lower-level health facilities (district hospitals, health centers, and dispensaries), and include other professional cadres such as pharmacists, dentists, laboratory scientists, and community healthcare workers to provide a broader picture of needs for health education and resources for SCD diagnosis and care in the whole country. Comparison of the level on knowledge on SCD among the various medical specialists on large study will also be of interest.

## Data Availability

The datasets presented in this article are not readily available because the primary researcher is still analyzing data for subsequent publications. Requests to access the datasets should be directed to AJ, ajonathan@blood.ac.tz.
